# Odor polymorphism in deceptive *Amorphophallus* species - a review: Odor polymorphism in *Amorphophallus*

**DOI:** 10.1080/15592324.2021.1991712

**Published:** 2021-11-28

**Authors:** Cyrille Claudel, Simcha Lev-Yadun

**Affiliations:** a Institute for Plant Science and Microbiology, Department of Biology, University of Hamburg, Hamburg, Germany; b Department of Biology & Environment, Faculty of Natural Sciences, University of Haifa-Oranim, Tivon, Israel

**Keywords:** Scent compounds, mimicry, polymorphism, evolutionary trends

## Abstract

Some plant lineages, such as Araceae and Orchidaceae, have independently evolved deceptive flowers. These exploit the insect’s perception and deceive the insects into believing to have located a suitable opportunity for reproduction. The scent compounds emitted by the flowers are the key signals that dupe the insects, guiding them to the right spots that in turn ensure flower pollination. Most species of the genus *Amorphophallus* of the Araceae emit scent compounds that are characteristic of a deceit, suggesting a specific plant pollinator interaction and according odors. However, only a few clear evolutionary trends in regard to inflorescence odors in *Amorphophallus* could be traced in previous studies – an intriguing result, considered the multitude of characteristic scent compounds expressed in *Amorphophallus* as well as the key function of scent compounds in deceptive floral systems in general. At least two factors could account for this result. (1) The deceptive pollinator-attraction floral system, including the emitted scent compounds, is less specific than assumed. (2) An evolutionary trend cannot be discerned if the intraspecific scent variation (odor polymorphism) exceeds the interspecific odor variation. Therefore, we discuss the potential deceptive function of the emitted scent compounds, in particular those that are related to cadaveric decomposition. Moreover, we review the data about emitted scent compounds in *Amorphophallus* with a focus on putative odor polymorphism. Upon examination, it appears that the emitted scent compounds in *Amorphophallus* are highly mimetic of decomposing organic materials. We show that several species display odor polymorphism, which in turn might constitute an obstacle in the analysis of evolutionary trends. An important odor polymorphism is also indicated by subjective odor perceptions. Odor polymorphism may serve several purposes: it might represent an adaptation to local pollinators or it might assumingly prevent insects from learning to distinguish between a real decomposing substrate and an oviposition-site mimic.

## Introduction

### Deceptive flowers

The art of deception is known to be practiced by thousands of plant species for the sake of avoiding herbivory,^1^^,2^ for seed dispersal^3^ and for pollination.^4–8^ Deceptions by plants are based on visual components, chemical ones, or on both. One of the most complex deceit types is sexual deception in Orchidaceae, which consists of both visual and olfactory mimicry of a specific female insect by a flower.^7–9^ Another deceit type that exploits the reproductive instincts of insects is brood-site mimicry or oviposition-site mimicry.

Oviposition site mimicry has independently evolved in several angiosperm plant families such as the Annonaceae, Apocynaceae, Araceae, Orchidaceae and Rafflesiaceae.^10,11^ The flowers or inflorescences visually and olfactorily mimic a specific substrate, which constitutes the main food source for saprophagous and copro-necrophagous insects and/or their larvae. The targets are deceived into believing to have located a suitable substrate for feeding, mating and/or breeding. Typical mimicked substrates are: carcasses, carrion, dung, feces, rotting plant material or mushrooms.^10^

The key communications signals in this type of plant-pollinator interaction are the scent compounds.^10,11^ Based on chemical mimicry, they are emitted to specifically exploit the insect’s perception.^10,11^ Moreover, scent compounds have a wide operational range, especially if they are promoted by heat, such as in thermogenic species.^12–14^

In the Araceae, oviposition-site mimicry is found in several genera from the Aroideae subfamily, the genus *Amorphophallus* among others.^15^ The plant-pollinator interactions within the Araceae are reported to be based on perception biases and not on co-evolution, the color and odor preferences of the visiting insects, beetles in particular are evolutionary conserved and the plants exploit preexisting preferences.^5,10,16–19^ The evolutionary conservation of preferences for olfactory signals such as methoxylated aromatic hydrocarbons, and by consequence animal perception, can be described as “variation around a theme”.^18^ The convergence between the scent chemistry of *Amorphophallus*, stapeliads and other brood site deceit flowers is likely to be based on sensory exploitation.^10,20,21^

However, only few authors investigated and actually tested the evolutionary relationship between innate preferences of pollinating insects and the emitted volatile organic compounds (VOCs) of the pollinated oviposition-site mimics; within one or even across convergent plant lineages.^8,10,16,18,22^ Moreover, it has been demonstrated that varied proportions of scent compounds can lead to different signaling functions.^23^ Furthermore, we emphasized the necessity to consider the pollinators and the herbivores when investigating the evolution of floral traits. Similarly, because carrion and dung odors are good predictors of three potential dangers to mammalian herbivores, namely pathogenic microbes, proximity of carnivores, and feces-contaminated habitats that present high risks of parasitism, it has been proposed that in addition to pollinator attraction, carrion and dung odors may repel mammalian herbivores.^24^

### Amorphophallus

The genus *Amorphophallus* Blume ex Decne comprises some 230 species^25–28^ and is the third largest genus in the Araceae family [^29^,onwards] as well as the largest Araceae genus with a paleotropical distribution. The genus has been delimited into four subgenera, namely the subgenera *Afrophallus* Hett. & Claudel, *Amorphophallus, Metandrium* and *Scutandrium* Hett. & Claudel.^30^

**Figure 1.**
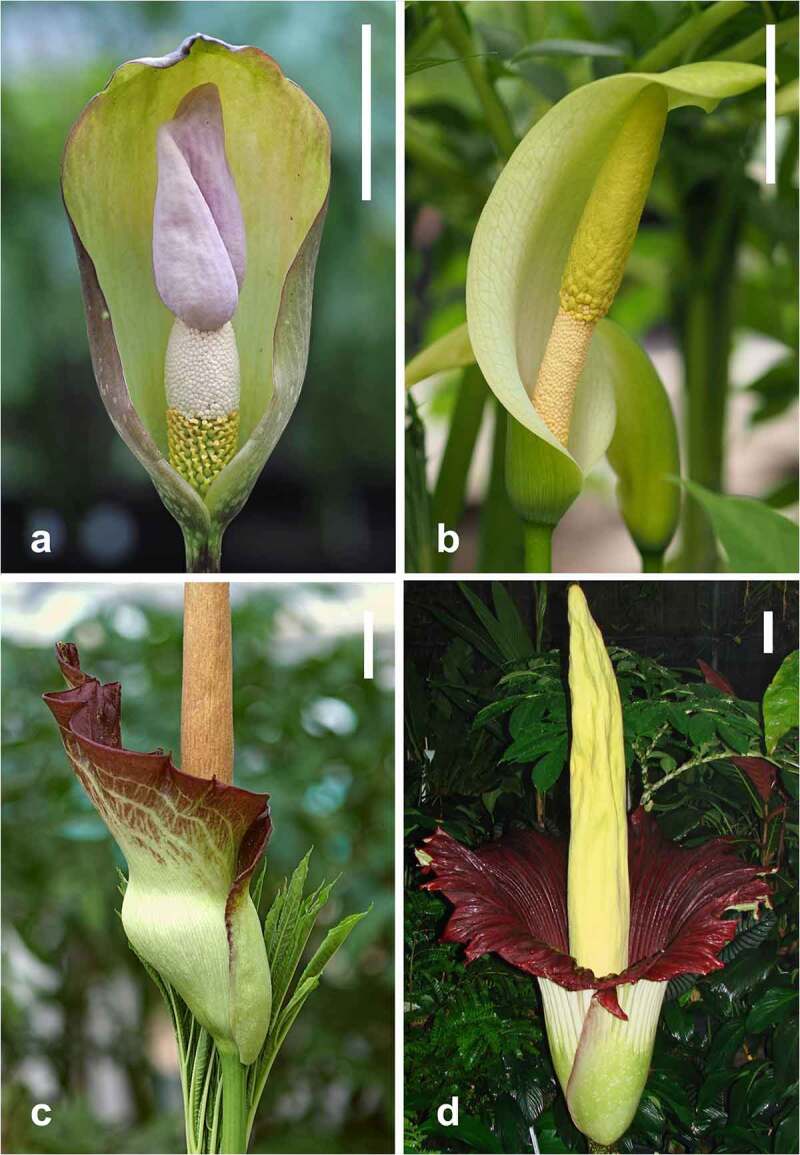
Inflorescences of A: *Amorphophallus thaiensis* and B: *A. albus*, consisting of a spadix surrounded by a spathe. C: spathe separated into a limb and a base forming a floral chamber in *A. angolensis*. The pistillate and the staminate flowers are freely accessible in A & B. D: Inflorescence of the iconic *A. titanum* the largest carrion flower of the genus. Scale bars: A, B & C = 5 cm. D = 10 cm. Photographs: A, B & C = Cyrille Claudel. D = Steve Jackson.

*Amorphophallus* inflorescences consist of a spadix and a spathe borne on a peduncle.^31^ In most species, the spathe is funnel or bowl shaped during anthesis and the spadix is freely accessible to insect visitors or pollinators (Figure 1a, b). More rarely, the spathe is constricted in a lower base (kettle) and an upper limb (Figure 1c). When strongly constricted, the kettle forms a floral chamber or a trap.^32^ The spadix has three zones (Figure 1a). The lowermost zone bears the female (pistillate) flowers, and the zone above it bears the male (staminate) flowers. The terminal zone, the appendix, essentially serves for attraction of pollinators through scent emission, sometimes enhanced through heat generation, such as in the iconic *A. titanum* (Figure 1d).^12,13,33^ Typical of the Araceae, *Amorphophallus* inflorescences are protogynous and anthesis usually lasts for two days. Stigma receptivity is signaled by the release of VOCs which serve to attract insect visitors and pollinators.

### *Identification and evolution of scent compounds in* Amorphophallus

The scent compounds of nearly a hundred species of the genus *Amorphophallus* have been identified^13,34–41^ (Supplemental material, Table S1). The scents emitted by *Amorphophallus* species are reminiscent of carrion, various forms of excrements, fish, sewerage, nauseating gases, rancid cheese, fermenting fruit and mushrooms.^34–41^ Table S1 lists all the investigated *Amorphophallus* species, the analyzed voucher and sampling time and the identified scent compounds in their relative amount as well as the subjective perception.

Kite and Hetterscheid^37,38^ identified the scent compounds of 92 *Amorphophallus* species using GC-MS [gas chromatography–mass spectrometry). They generated seven main categories based on the chemical identity of the defining scent compound per *Amorphophallus* species, mapped these as characters onto the Bayesian consensus tree from ^30^ and investigated the evolutionary trends of inflorescence odors in *Amorphophallus*. However, the inferred trends provided a heterogeneous picture ^38^, which is an intriguing result, considering that scent compounds are assumed to be the key elements of deceptive floral systems.^10,11,21,23,42,43^

^38^ found that dimethyl oligosulphides are released in species across all four subgenera and are the most common constituents in half of the 92 species studied. Dimethyl oligosulphides are characteristic of the decomposition of various organic matters, ranging from sulfur-rich vegetables, to cancerous wounds and most importantly cadaveric decomposition and carnivore dung.^10,41,44^ They are released in various plant lineages and represent well-known attractants for various copro-necrophagous beetles and flies.^45–47^ Furthermore, two distantly related *Amorphophallus* clades, comprising a handful of species each, were found to be characterized by the emission of benzenoid compounds, which are considered to be strongly evolutionarily constrained.^38^ Moreover, several smaller phylogenetic subunits comprising a few closely related species were identified, such as the *A. aphyllus* group, sharing a similar inflorescence morphology and similar odor types, namely dung odors.^38^

In contrast, other odor types were found to have a high degree of plasticity, evidenced by the observation that some sister species release different scent types.^38^ For example, two closely related Asian species with fungal odors, *A. obscurus* and *A. polyanthus* emit chemically very different scent compounds. *Amorphophallus obscurus* releases high proportions of various alcohols, such as isoamyl alcohol whereas *A. polyanthus* mainly emits a ketone, more precisely 2-heptanone. Similarly, *A. ongsakulii* and *A. myosuroides* are characterized by the emission of 90% 2-nonanol and 75% α-ketoisocaproic acid respectively. Consequently, although closely related, both species are placed in two different categories sensu ^38^. Moreover, a third closely related species, *A. sumawongii*, is characterized by the emission of dimethyl oligosulphides. However, at least this species is also morphologically different.^38^ Lastly, the two African mainland sister species *A. abyssinicus* and *A. mossambicensis* share a similar inflorescence morphology; however, *A. mossambicensis* is a member of the aliphatic esters group and smells of carrion whereas *A. abyssinicus* smells of dung and belongs to the terpenoids and alkanes group.^38^

Beyond that, similarly to previous phylogenetic studies,^48–50^ no characters could be identified that would circumscribe larger phylogenetic units. ^38^,proposed that variation in pollinator taxa is the driving force, leading to the divergence of odor types in some species as well as to the convergence of some odor types in others.

However, besides specific olfactory cues, such a specialized plant-pollinator interaction may also have to rely on an evolutionary constrained inflorescence morphology, discriminating between different insect types. This appears to be unlikely as the spathe of many *Amorphophallus* species forms a funnel- or bowl-like structure (Figure 1), which is easily accessible for a large insect array. Some *Amorphophallus* species, such as *A. ongsakulii* or *A. interruptus* have small and frail inflorescences or a very tight spathe entrance, which excludes pollinators of a larger size. However, beyond that, there seems to be few further discriminatory traits, especially if compared to highly constrained flowers of another deceit type, namely sexual deception in orchids.^7–9^ What is more, the apparent olfactory deceit of the majority of *Amorphophallus* species is based on the emission of dimethyl oligosulphides, which is not indicative of a specific plant pollinator interaction as these volatiles attract a wide array of insects searching for decomposing organic matter for feeding, mating or breeding. Unfortunately, the pollinators of *Amorphophallus* species are largely unknown, making it impossible to investigate this putative relationship on a larger scale.^51^ Insect pollinators or visitors have been reported for little more than 20 *Amorphophallus* species and roughly a third of these observations rely on a single inflorescence per species.^51^ Moreover, most observations suggest an unspecialized plant-pollinator interaction or at least the attraction of a multitude of different insects or other arthropods.^51^ Nonetheless, beetles appear to be the main pollinator group in most investigated species.^51,52^

That said, there is another hypothesis possibly accounting for the several trends in *Amorphophallus* inflorescence odors that have not been considered yet, which is intraspecific scent variation or odor polymorphism. Investigations of the scent emissions in the genus *Arum*, another and better studied member of the subfamily Aroideae, revealed that the emitted scent compounds can vary considerably within a single species. Significant differences in the proportion of the emitted scent compounds were detected in *Arum italicum* and *A. maculatum*.^39,53–55^ In some cases, the differences in the spectrum of the emitted scent compounds were so significant that they were categorized as chemotypes.^54^ Another study investigated the inflorescence morphology, pollinators and scent compounds in natural hybrids, originating from parental populations from *Arum italicum* and *A. maculatum*.^56^ Remarkably, eight scent compounds that were not detected in the floral odor of either parental species were detected in the hybrid offspring.^56^ Odor polymorphism has also been documented in the deceptive orchid *Dactylorhiza romana*.^17^

## Objectives

The aim of the present review is twofold. (1) Decomposition of organic matter, be it vegetable matter, carrion or dung, appears to represent the dominant deceit type in the genus *Amorphophallus* [e.g., ^38^]. In order to ascertain their deceptive function, several of the scent compounds emitted by the model, cadaveric decomposition in particular, are compared to the scent compounds emitted by *Amorphophallus* carrion mimics. (2) Assuming that intraspecific odor polymorphism in *Amorphophallus* is as important as in the genus *Arum*, it is likely to shade putative evolutionary relationships and trends in regard to inflorescence odors. Consequently, the relevant literature is reviewed in regard to odor polymorphism in *Amorphophallus*.

### Scent compounds emitted by cadaveric decomposition

Volatile organic compounds (VOCs) released after death are described as the chemistry of death or thanatochemistry.^57^ The decomposition of a cadaver is initiated by the degradation of the body through its own enzymatic and chemical reactions, defined as autolysis. The breakdown of four major biological molecule classes during the various stages of decomposition is at the base of the resulting mixture of volatile organic compounds, i.e., the scents of death. The classes are: proteins, nucleic acids, lipids and carbohydrates, and their degradation ultimately leads to VOCs such as diamines, sulfur compounds (dimethyl oligosulphides), phenolic molecules such as indole and skatole, organic acids, alcohols, ketones, aldehydes, esters and ethers, hydrocarbons, nitrogen and oxygenated compounds such as acetone.^57^ Conversely, the emitted compounds inform the insects about the nutritive potential of the decomposing organic material, since decomposing lipids will lead to different signals from proteins, etc.^10^ However, not only the nutritive composition but also the stage of decomposition is indicated by the emitted volatiles.^10^

Once internal microorganisms take the lead, bloating marks the beginning of putrefaction. The environment and abiotic parameters such as temperature, humidity, and oxygen concentrations can have a strong influence on microorganismal activity, and therefore on decomposition itself. Following ^57^, autolysis and putrefaction can be subdivided into five general stages; fresh, bloated, active decay, advanced decay and skeletonization. ^58^ investigated the human decomposition fluid formed during autolysis and corpse putrefaction, in order to identify the most characteristic scent compounds for a cadaver-detection dog-training program. ^58^, identified 35 VOCs found in 95% of all analyzed samples, among others: dimethyl trisulfide, which after dimethyl disulfide constitutes the most abundant compound across the genus *Amorphophallus*, and pyrazine, the defining volatile compound of *A. preussi*.^38^ Furthermore, ^58^, identified 2-decanone, hexanal, nonanal, phenol and 2-undecanone, all being minor volatile compounds in different *Amorphophallus* species,^38^ and also 2-heptanone, the major scent compound in *A. polyanthus*, as well as propionic acid, which is present in *A. gigas*.^36,38,^also identified acetone in 88% of the analyzed samples, a compound also released by *A. borneensis* (8%), *A. commutatus* (11%), *A. erythrrorhachis* (12%), *A. konjac* (2% + 6%), *A. plicatus* (2%), *A. macrorhizus* (3%), *A. henryi* (7%), *A. eburneus* (18%) and *A. tinekeae* (9%).^58^

Similarly, ^59^,analyzed the profile of VOCs released by pig carcasses during the first 75 hours after death. Dimethyl oligosulphides were identified, notably ethyl acetate, which is the major volatile component of *A. antsingyensis* and *A. consimilis*. Furthermore, 1-propanol and 3-methyl-1-butanol, two of the major volatile compounds for the *Amorphophallus* group defined by high proportions of aliphatic alcohols and ketones^38^ were identified. ^57^,also investigated the decomposition of pig carcasses and recovered 104 volatile organic compounds, amongst them trimethylamine, the defining scent compound of the nitrogen-containing *Amorphophallus* group,^38^ together with 4-methylpentanoic acid (isocaproic acid) and butanoic acid, the defining scent compounds of the *Amorphophallus* group defined by high proportions of aliphatic acids.^38^ Without comparing every scent compound of the models and the mimics one by one, it becomes apparent that there is a remarkable overlap between the single scent compounds emitted by human decomposition fluid formed during putrefaction, pig carcasses and various *Amorphophallus* species (Table 1). Therefore, referring to the first objective of this review, it is reasonable to assume that the function of these scent compounds is mimicking cadaverous decomposition.

**Table 1.** Scent compounds released by human decomposition fluid, pig carcasses and *Amorphophallus* species. Numbers refer to: **1**) ^58^, **2**] ^59^, **3**) ^57^. Except for *A. gigas*,^36^ all scent compounds emitted by *Amorphophallus* species are retrieved from Kite and Hetterscheid.^37,38^ Group defining compounds refer to compounds used by ^38^,to categorize major scent groups

**Selected VOCs emitted during cadaveric decomposition**	**ref.**	**also emitted by the *Amorphophallus* species (rel. % of the total odor composition in descending order)**	**used as group defining compound**^38^**of the:**
1-phenylethanone (acetophenone)	1	*A. symonianus* (60%), *A. amygdaloides* (60%), *A. cicatricifer* (55%, 39%), *A. pulchellus* (5%), *A. putii* (2%), *A. yuloensis* (11%, 6%)	benzenoid compounds group
1-propanol	2	*A. cirrifer* (16%, 11%), *A. obscurus* (10%), *A. pilosus* (7%)	aliphatic alcohols and ketones group
2-decanone	1	*A. ankarana* (2%)	
2-heptanone	1	*A. polyanthus* (85%, 62%), *A. eichleri* (30%, 25%), *A. ankarana* (3%)	aliphatic alcohols and ketones group
2-undecanone	1	*A. ankarana* (3%)	
3-methyl-1-butanol	2	*A. ankarana* (39%), *A. cirrifer* (36%, 16%), *A. henryi* (30%, 7%), *A. obscurus* (21%), *A. borneensis* (8%), *A. commutatus* (3%), *A. konjac* (3%)	aliphatic alcohols and ketones group
4-methylpentanoic acid	3	*A. elatus* (100%), *A. atroviridis* (98%), *A. linearis* (94%), *A. macrorhizus* (97%, 95%), *A. angustispathus* (50%), *A. saraburiensis* (23%), *A. scutatus* (7%), *A. baumannii* (6%), *A. johnsonii* (4%),	aliphatic acids group
acetone	1	*A. eburneus* (18%), *A. erythrrorhachis* (12%), *A. commutatus* (11%), *A. tinekeae* (9%), *A. borneensis* (8%), *A. henryi* (7%), *A. konjac* (6%, 2%), *A. macrorhizus* (3%), *A. plicatus* (2%)	
butanoic acid	3	*A. taurostigma* (74%), *A. saraburiensis* (4%), *A. scutatus* (4%)	aliphatic acids group
dimethyl oligosulphides	2	identified in varied proportions in **58 out of 92** investigated *Amorphophallus* species	sulfur-containing compounds group
dimethyl trisulfide	1	identified in varied proportions in **47 out of 92** investigated *Amorphophallus* species	sulfur-containing compounds group
ethyl acetate	2	*A. consimilis* (77%, 57%), *A. haematospadix* (65%), *A. annulifer* (60%), *A. antsingyensis* (43%), *A. laoticus* (23%), *A. borneensis* (10%), *A. baumannii* (5%), *A. henryi* (2%)	aliphatic esters group
hexanal	1	*A. pilosus* (3%)	
nonanal	1	*A. elliottii* (3), *A. eburneus* (3%), *A. erythrorrhachis* (2%)	
phenol	1	*A. impressus* (6%)	
propionic acid	1	*A. gigas* (4%)	
pyrazine	1	*A. preussi* (61%)	nitrogen-containing compounds group
trimethylamine	3	*A. brachyphyllus* (85%), *A. eburneus* (64%), *A. tinekeae* (35%), *A. angolensis* (18%), *A. plicatus* (13%), *A. longispathaceus* (4%), *A. konjac* (2%)	nitrogen-containing compounds group

### *Investigation of odor polymorphism in* Amorphophallus

Kite and Hetterscheid^37,38^ analyzed 15 *Amorphophallus* species twice and four species thrice (Table S1). Some species, such as *A. macrorhizus, A. mossambicensis, A. paeoniifolius* and *A. sumawongii*, yielded more or less similar scent compound spectra in all analyses, although different individuals were investigated and compared. *Amorphophallus consimilis, A. variabilis* and *A. yuloensis* were also analyzed twice and showed similar results. However, similar results should be expected here since clonally propagated plants had been analyzed. The documented variation can obviously at least partly be accounted for by different study methodologies^37,38,40^ or because of different sampling times or sample overloads, etc.^38^ Particularly, the sampling time seems to be a critical aspect, as the variation in scent composition may strongly vary during anthesis.^13,38,41,55^ Thus, whenever possible, a consistent sampling protocol was ensured, minimizing the influence of the sampling time.^38^ However, some individuals reveal a broader intraspecific variation or scent polymorphism.

Table 2 shows the *Amorphophallus* species that have been analyzed repeatedly and which show the most significant differences between the analyzed individuals. The three analyses of *A. konjac* also showed significant differences (Table 2; ^37,38^). Even more so, if compared to the analysis of *A. konjac* by ^34^,(Table S1).

**Table 2.** Some selected *Amorphophallus* species which show significant odor polymorphism. Differing scent classes within a species are highlighted in bold. If specified in the original publications, voucher and/or origin are provided. The quantity of the identified scent compounds is presented as in the original publications, either as percentage or as symbol (x; +; -). Percentage numbers are rounded in two cases.^34,40^ References are given as numbers and refer to: **1**) ^37^, **2**) ^13^, **3**) ^41^, **4**) ^34^, **5**) ^38^, **6**) ^40^

species & voucher	chem. category sensu Kite & Hetterscheid	scent compounds per species and individual in % or as given in according reference.									
		isoamyl alcohol	isoamyl acetate	β-pinene	tridecane	α-pinene	camphene	skatole	2-butanol	acetone	butyl acetate
*A. henryi* HAM 270	alcohols and ketones	30		25	22	16	2	2			
*A. henryi* 1994–3573	alcohols and ketones	7	18	10	6	2			17	7	6
		1-phenylethanone	methyl cinnamate	1-phenylethyl acetate							
*A. symonianus* HAM 924	benzenoid compounds	60	39								
*A. symonianus* 1998–3421	benzenoid compounds		6	89							
		dimethyl disulfide	dimethyl trisulfide	dimethyl tetrasulphide	2-heptanone	indole	phenylethylalcohol	butyl heptanoate	2-pentanone	α-ketoisocaproic acid	1-butanol
*A. eichleri* not specified	sulfur compounds	62	15	1	7	2	1				
*A. eichleri* 1994–7554 (1)	sulfur compounds	56	7	1	25			8			
*A. eichleri* HAM 007 [2)	**alcohols and ketones?**	23	1		**30**				13	10	6
		dimethyl disulfide	dimethyl trisulfide	dimethyl tetrasulphide	ethanol	2/3-methyl-2-butenal	trimethylamine	3-methyl-1-butanol	acetaldehyle	acetone	2-butanone
*A. konjac* not specified	sulfur compounds	76	17								
*A. konjac* 1997–111	sulfur compounds	55	3		9	6	6	3	3	2	2
*A. konjac* HAM 168	sulfur compounds	40	17	1		3	2	3		6	12
*A. konjac* China, KBG	sulfur compounds	43	26	2							
		dimethyl disulfide	dimethyl trisulfide	dimethyl tetrasulphide	dimethyl pentasulphide	1-butanol	isocaproic acid	4-methyl-1-pentanol	butanoic acid	ac s-methyl thioester	acetic acid
*A. scutatus* HAM 589	sulfur compounds	34	61	5							
*A. scutatus* HAM 590	sulfur compounds	18	29	11	1	12	7	5	4	2	2
		dimethyl disulfide	dimethyl trisulfide	dimethyl tetrasulphide	ac s-methyl thioester	pungent smell	sweet smell	almond like	benzaldehyde	trimethylamine	3-methyl-butanal
*A. titanum* not specified	sulfur compounds	75	10								
*A. titanum* 1997–5514	sulfur compounds		25	1	3						
*A. titanum* Palm Garden	**benzenoid compounds?**					**x**	**x**	**x**	**x**		
*A. titanum*: gas sample	**nitrogen-containing?**		+							**++**	+
*A. titanum*: appendix	**aliphatic acids?**								4

**Table 2.** continue

species & voucher	scent compounds per species and individual in % or as given in according reference.													Ref.
	isobutyl acetate	undecane	caryophyllene	ethyl acetate	limonene	3-methyl-2-hexanone								
*A. henryi* HAM 270														**5**
*A. henryi* 1994–3573	6	5	2	2	2	1								**5**
*A. symonianus* HAM 924														**5**
*A. symonianus* 1998–3421														**5**
	2-hexanone	4-methyl-1-pentanol	2-pentanol											
*A. eichleri* not specified														**1**
*A. eichleri* 1994–7554 (1)														**5**
*A. eichleri* HAM 007 [2)	3	2	2											**5**
	2-methyl-1-butanol	isoamyl alcohol	β- caryophyllene	3-methyl-2-pentanone	4-hydroxy-4-methyl- 2-pentanone	n- nonaldehyde	butyl ether	3-methyl-1-pentanol	n-dodecane	2- pentanone	n-tridecane	butyl 2- propenoate		
*A. konjac* not specified														**1**
*A. konjac* 1997–111														**5**
*A. konjac* HAM 168	2													**5**
*A. konjac* China, KBG	3	6	3	2	2	2	2	2	1	1	1	1		**4**
	ethyl acetate	2-methylbutanoic acid												
*A. scutatus* HAM 589														**5**
*A. scutatus* HAM 590	2	2												**5**
	methyl thiolacetate	acetic acid	isovaleric acid	isovaleric acid	butyric acid	benzylalcohol	γ- butyrolactone	3-hydroxy-2- butanone	2- phenoxyethanol	phenol	4-hydroxy-4-methyl-2-pentanone	nonanal	trimethyl pyrazine	
*A. titanum* not specified														**1**
*A. titanum* 1997–5514														**5**
*A. titanum* Palm Garden														**2**
*A. titanum*: gas sample	-	+	-											**3**
*A. titanum*: appendix				**22**	**17**	16	12	6	3	3	3	2	2	**6**

**Table 3.** *Amorphophallus* species which show significant odor polymorphism based on the subjective human scent perception, with according reference

species	subjective odor perception as described in according reference	reference
*A. aphyllus*	dung	^30^
*A. aphyllus*	fruity, melon-like, with added vodka	[pers. commun. S. Jackson]
*A. cicatricifer*	gaseous plus fruity	^37^
*A. cicatricifer*	gaseous, almonds	^38^
*A. commutatus*	dead meat	^38^
*A. commutatus*	rottening meat	^63^
*A. commutatus* var. *anmodensis*	gaseous, fruity	^63^
*A. commutatus* var. *anshiensis*	gaseous, fruity	^63^
*A. commutatus* var. *wayanadensis*	rottening meat	^63^
*A. fallax*	gaseous	^37^
*A. fallax* [1]	gaseous, sweet	^38^
*A. fallax* [2]	gaseous, sweet	^38^
*A. gigas*	spoiled meat	^61^
*A. gigas*	rotten, fishy, sour	^36^
*A. johnsonii*	sewerage	^38^
*A. johnsonii*	carrion	^64^
*A. konkanensis*	cheese	^38^
*A. konkanensis*	rottening meat	^63^
*A. mossambicensis* [1]	carrion	^38^
*A. mossambicensis* [2]	carrion	^38^
*A. mossambicensis* [3]	acidic, dung	^38^
*A. prainii*	gaseous	^37^
*A. prainii*	rotten meat	Soepadmo, ^65^1973
*A. sylvaticus*	bad vegetables	^38^
*A. sylvaticus*	rottening meat	^63^
*A. symonianus*	fruity, cinnamon, shoe polish	(personal obs.]
*A. symonianus* [1)	almond, chemical	^38^
*A. symonianus* [2]	almond, chemical	]^38^
*A. titanum*	gaseous plus urine	^37^
*A. titanum*	gaseous, rotting vegetables	^38^
*A. titanum*	carrion and weakly sweet	^13^
*A. titanum*	old fish	^61^
*A. titanum*	rotting flesh, changing to excrement	^66^
*A. titanum*	decayed cabbage, garlic and pungent sour	^35^
*A. titanum*	nearly scentless	^60^
*A. titanum*	strong scent	^60^
*A. titanum*: appendix sample	rotting meat	^40^
*A. titanum*	slight rotten fruit like, yellow pickled radish, rotten egg, rotting animal-like, rotten fish, rotten egg	^41^

Likewise, a significant variation was detected in the two specimens of *A. scutatus* (Table 2). The scent of the first individual consists of 100% dimethyl oligosulphides, whereas the scent of the second individual contains dimethyl oligosulphides (59%), 1-butanol (12%), 4-methyl-1-pentanol (5%), butanoic acid (4%), S-methyl thioesters (2%), acetic acid (2%), ethylacetate (2%), isocaproic acid (7%), and 2-methylbutanoic acid (2%).^38^

Particularly two of the three analyses of *A. eichleri* are of interest, insofar as the major compound was not the same in analysis one (56% dimethyl disulfide) and in analysis two (30% 2-heptanone) [^38^, Table 2]. Therefore, the second individual of *A. eichleri* could be categorized under “alcohols and ketones” group instead of the “sulphur compounds” one.^38^

However, the most remarkable differences are found between the different analyses of *A. titanum*.^13,35,37,38,40,41^ The comparison of the results must be done cautiously, as different sampling and analysis methods have been employed. Particularly the methodological approach from ^40^ differs strongly. Nevertheless, some differences are noteworthy. ^37^, identified dimethyl disulfide (75%) and trimethyl disulfide (10%) as the major volatile compounds in *A. titanum* and described the scent as gaseous plus urine (Table 2). In a second analysis ^38^, identified dimethyl disulfide (70%), trimethyl disulfide (25%), tetra disulfide (1%) and S-methyl thioesters (3%) and described the scent as gaseous or as rotting vegetables (Table 2).

^41^,identified dimethyl trisulfide as the major component; moreover, they identified various compounds not detected by ^38^. Furthermore, ^41^ closely followed anthesis of *A. titanum* by the human nose, and the scent changed over time from “slight rotten-fruit-like odor” to “yellow-pickled-radish, rotten-egg, rotting-animal-like odour”, then “strong rotting-animal-like odor” and finally “rotten-fish and rotten-egg” (Table 2). The scent composition obviously varies strongly during anthesis. Based on the results of^35,41^,attempted to objectively describe the scent compounds of *A. titanum* using electronic noses, based on semiconductor-sensors. They compared the odor profile of an *A. titanum* plant grown in Kagoshima with the odor profile of the *A. titanum* plant grown in Tokyo that was previously studied by ^41^. ^35^ described the odor profile from *A. titanum* as “decayed cabbage, garlic and pungent sour”.

In contrast, no sign of dimethyl disulfide and dimethyl trisulfide could be detected in the analysis of *A. titanum*.^13^ Moreover, the initial carrion smell changed to a weak sweet smell during anthesis. Benzaldehyde, an almond-like smelling compound, was identified as the dominant compound during the ongoing of anthesis.^13^ The analyzed plant descended from material cultivated in the Palm Garden in Frankfurt/Main, Germany and had been originally collected near Padang in Indonesia.

Another investigation of the scent chemistry of *A. titanum* was performed by ^40^. However, it belonged to another plant source, i.e., Dr. Louis Ricciardiello, New Hampshire, USA. A total of 25 scent compounds were identified in this case, and the resulting odor profiles were again different [^40^, Table 2]. No dimethyl- di- or trisulfides were identified. Instead, the three major scent compounds emitted by the appendix were isovaleric acid (21.6%), butyric acid (17.0%) and benzyl alcohol (16.2%) (Table 2). However, the methodological approach followed by ^40^,differed significantly in that the analyzed tissues were cut off the plant and pre-treated.

Additionally, it should be noted that ^60^ examined two flowering *A. titanum* individuals. These two plants were the first ones to flower on the European continent and their development was closely followed.^60^ One plant was found to be strongly scented whereas the second inflorescence was found to be nearly scentless.^60^

Only the two analyses from Kite and Hetterscheid^37,38^ on the one hand, and the two analyses from ^41^,and ^35^,on the other, yielded a similar odor profile for *A. titanum*. It was not specified if Kite and Hetterscheid^37,38^ repeatedly analyzed the same plant, or two different plants. In any case, if two different plants were analyzed, they are likely to have the same origin since at that time only a few clones of *A. titanum* were shared among different botanical gardens. As for the plants analyzed by ^41^,and ^35^, they both originated from one infructescence, harvested in 1993.^61,62^ These plants are therefore unequivocally of the same maternal origin and a similar odor profile could be expected.

Therefore, the odor profiles from all analyzed *A. titanum* plants, except from those of the same genetic origin, are markedly different.^13,35,37,38,40,41^ Thus, at least in the case of *A. titanum*, the odor profiles of single specimens only partially reflect the genetic and olfactory variability of the species. Moreover, if categorized per major scent compounds, these plants would not be categorized under “sulphur compounds”^37,38^ but under benzenoid compounds,^13^ nitrogen-containing compounds,^41^ and under aliphatic acids.^40^ Thus, *A. titanum* could be placed in four different scent categories sensu ^38^.

The scent experience based on human perception also indicates significant variation in *A. titanum* and in several other species (Table 3). Although subjective, the differences are too important to be ignored. Some species, such as *A. cicatricifer* and *A. fallax*, show slight differences in their odor profiles (Table 3). More important, however, is the perceived odor variation within the subspecies of *A. commutatus*, which range from “rottening meat” to “gaseous and fruity”.^63^ Likewise, one individual of *A. gigas* was perceived as smelling like “spoiled meat”,^61^ whereas another has been described as smelling “rotten, fishy and sour”.^36^ Furthermore, *A. maximus* and *A. mossambicensis* can smell like “rotting meat” or “dung”,^37,38^ whereas *A. konkanensis* is either reminiscent of “cheese”^38^ or of “rottening meat”.^63^ Furthermore, the scent of *A. sylvaticus* has been described as “rottening” meat by ^63^,and as “bad vegetables” by ^38^. The scent of two specimens of *A. symonianus* has been described as “almond, chemical” by ^38^. However, some specimens of *A. symonianus* also smell fruity and strongly cinnamon-like with a pinch of shoe polish (personal observation). Strikingly, the scent of *A. aphyllus*, a species that is known as a dung species par excellence^30,38^ has recently been described as “fruity, melon-like, with added alcohol” [Steve Jackson, pers. comm.]. Apparently, the olfactorily deceit in *A. aphyllus* ranges from dung to fermenting fruit.

### From carrion to sweet odors

^38^,also sampled two individuals of *A. symonianus* that showed a strong difference in the emitted quantities of two aromatic compounds or benzenoids, 1-phenylethylacetate and 1-phenylethanone. The scent of plant one consisted mainly of 1-phenylethanone (60%) and the scent of plant two, of 1-phenylethylacetate (89%) (Table 2). Disregarding the difference in scent composition between the two specimens, the odor is composed of only a few scent compounds.^38^ Two questions emerge from these finds. First, how do sweet odor types fit into the variation around a theme revolving around decomposition and decay? Second, is the number of contributing scent compounds indicative of the relationship between the plant and its pollinators? In essence, does a scent composition that comprises exclusively one or two scent compounds indicate a more specialized plant-pollinator interaction than a scent composition that comprises 10–20 scent compounds? One further VOC identified in 95% of all samples of human decomposition fluid was 1-phenylethanone or acetophenone.^58^ Acetophenone is the simplest aromatic ketone, and interestingly, the major scent compound of one of the *A. symonianus* individuals. It is also a major scent compound in *A. amygdaloides* and *A. cicatricifer*, and a minor scent compound in *A. pulchellus, A. putii*, and *A. yuloensis*.^38^ Although speculative, it is conceivable that these species just mimic an earlier and sweet-scented phase of decomposition and/or target a different pollinator group as suggested by ^38^.

However, another well-supported clade in the subgenus *Metandrium* contains five species that, except for *A. amygdaloides* (see above), emit a scent that is entirely composed of 1-phenylethyl acetate.^38^ The species are *A. dunnii, A. putii, A. thaiensis* and *A. yunnanensis*, and the scent is reported to be generally perceived as fruity, or in the case of *A. dunnii, A. putii* and *A. yunnanensis* as reminiscent of grated carrots.^38^ This scent compound cannot be related to cadaveric decomposition and is not reported to be a known attractant otherwise. However, unfortunately the pollinators of all the mentioned species are completely unknown.

Similarly, there is another clade containing sweet-scented species of the subgenus *Scutandrium*, such as *A. albispathus, A. longituberosus* and *A. tenuispadix*, and these species emit anise-like odors almost solely based on 4-methoxyphenethyl alcohol as well as a minor addition of methyl 4-methoxybenzoate.^37,38^ It is unclear if and how these scents fit into the theme, as at least 4-methoxyphenethyl alcohol does not seem to be linked to cadaveric decomposition processes. Nonetheless, it is known that methoxylated aromatics in general, and 4-methoxyphenethyl alcohol in particular, are strong attractants to various beetle taxa.^67–69^

Apparently, species that emit benzenoid compounds have little variation if any, in their odor profiles.^38^ This suggests an evolutionary trend, linked to a specific pollinator.^38,70^ However, it must also be taken into account that only a handful of species, almost exclusively emit either 4-methoxyphenethyl alcohol or 1-phenylethanol derivatives each. Moreover, the species within both clades are closely related and the morphological variation between the species is low in both clades.^71,72^ For example, *A. putii* and *A. yunnanensis* from subgenus *Metandrium* are morphologically hardly distinguishable, the main difference being that the appendix in *A. putii* is laterally compressed.^71^ Likewise, the overall inflorescence morphology is identical in *A. albispathus, A. longituberosus* and *A. tenuispadix*; the defining characters of the species refer to the leaf architecture, the tuber shape and the pores of the anthers.^71^ Moreover, they all occur in Thailand.^71^ Consequently, both clades might represent starting points of speciation that putatively exploit another olfactory preference of Coleoptera.

## Discussion and conclusions

Obviously, the documented intraspecific variation in odor composition can be partly attributed to differing methodological approaches and/or to different sampling times.^38^ However, it might not fully account for the highlighted differences between individuals of several species. Furthermore, although subjective, it appears legitimate to address odor polymorphism, considering the varied odor characterizations in several *Amorphophallus* species.

Most species of two smaller and not closely related clades, the clade containing *A. albispathus, A. longituberosus* and *A. tenuispadix* from subgenus *Scutandrium* and the clade containing *A. putii, A. symonianus* and *A. yunnanensis* from subgenus *Metandrium*, exclusively emit benzenoid compounds, 4-methoxyphenethyl alcohol or 1-phenylethanol derivatives. These species have little or no variation at all in their scent composition. Moreover, except for acetophenone, these benzenoid compounds cannot be related to decomposition.

That said, the majority of *Amorphophallus* species emit more complex odor compositions that can be specifically related to decomposition processes. The emitted scent compounds perfectly mimic their natural decomposing counterparts and some species show a significant odor polymorphism. This particularly applies to *A. titanum*, where practically each analysis yielded a different odor spectrum. A high degree of odor polymorphism, as documented in *A. titanum* may blur the study of evolutionary trends when the intraspecific variation exceeds the interspecific variation in several species. Consequently, although the presented differences are only indicative, they nonetheless demonstrate the need for a more extensive and systematic sampling.

Moreover, odor polymorphism may serve several purposes that need to be addressed. Odor polymorphism might represent an adaptation to local variations in the entomofauna.^73^ Consequently, specimens of different geographical origins, ideally covering the full geographic distribution and/or the morphological range, should be investigated, in order to identify the whole odor profile of an *Amorphophallus* species. This would allow investigation of the correlation between scent composition and the local entomofauna. Moreover, if the full repertoire of emitted scent compounds of each species is known, finer boundaries between species or species groups might be revealed and more subtle evolutionary trends might be detected.

Alternatively, odor polymorphism might prevent insects from learning how to distinguish between the real decomposing substrate and the mimic.^74^ Carrion, dung and the like are subjected to several abiotic parameters. Moreover, decomposition processes are strongly influenced by the action of various microorganisms and never smell 100% identical.^57^ Consequently, variation in odor composition might in itself be evolutionarily constrained. Under this scenario it would be challenging to trace evolutionary trends based on major scent classes in odor composition, as variation in itself would constitute a trend. Variation would then constitute a form of speciation. Moreover, it might also imply a lower evolutionary constraint of floral traits that are related to the deceptive floral system, leading to increased morphological variation. Although assumptive, the observed intraspecific odor variation is important enough to consider and investigate this phenomenon.

From a functional point of view, it becomes evident that more detailed studies are required in order to better understand the reproductive strategies of *Amorphophallus* species. The visiting and pollinating insects need to be observed and documented. Moreover, when investigating the evolution of floral traits, the necessity not only to consider the pollinators but also the herbivores has been emphasized and in the case of *Amorphophallus*, the putative repellence of mammalian herbivores through carrion or dung odors should be investigated.^24^ Similarly, the simultaneous attraction of predators, preying on visiting insects needs to be considered too.^51^

## Supplementary Material

Supplemental Material
