# Supplementary material for: Odor polymorphism in deceptive Amorphophallus species - a review: Odor polymorphism in Amorphophallus
Source: Plant Signal Behav. 2021 Nov 28;16(12):1991712. doi: 10.1080/15592324.2021.1991712 (PMC9208769; doi:10.1080/15592324.2021.1991712)
Supplement: Supplemental Material [file KPSB_A_1991712_SM9241.docx]

Table S1: *Amorphophallus* species, their identified scent compounds and the according reference. The identified scent compounds and their relative amount per species in percent are provided in descending order. If specified in the original publications, voucher or origin as well as sampling time are indicated. Furthermore, the subjective scent characterisation based on human perception is included. S-methyl thioesters (Ac, Pr, Bu, iBu) are referenced as in Kite and Hetterscheid (2017). The quantity of the identified scent compounds is presented as in the original publications, either as percentage or as symbol (x ; +; -), indicating the presence and the quantity of a given compound.

| **Species** | **Voucher or origin** | **Human perception** | **Sampling time** | **Scent compounds** | **Reference** |
| --- | --- | --- | --- | --- | --- |
| *A. aberrans* Hett. | HAM 861 | foul, spicy, sewerage | 20:30-22:45 | 48% ac s-methyl thioester, 20% dimethyl disulphide (DMDS), 16% pr s-methyl thioester, 4% dimethyl trisulphide (DMTS), 4% methyl propanoate, 2% ibu s-methyl thioester, trace elements: dimethyl monsulphide (DMMS), dimethyl tetrasulphide (DM4S) | Kite & Hetterscheid, 2017 |
| *A. abyssinicus* (Rich.) N. E. Br. | HAM 1141 | cow dung | 17:30-20:30 | 25% 2,7-dimethyl-1,7-octadiene, 19% 3,7-dimethyl-2-octene, 12% 3,7-dimethyl-1-octene, 11% 2-methyl-1-butanol, 3% 2-butanone, 2% β-selinene, 3% unk. sesquiterpene, various sesquiterpenoids | Kite & Hetterscheid, 2017; 21 |
| *A. albispathus* Hett. | unspecified | anise-like | 07:00-09:00 | 99% 4-methoxyphenethyl alcohol | Kite & Hetterscheid, 1997 |
| *A. albus* P.Y .Liu & J.F.Chen | unspecified | gaseous | 08:00-09:00 | 59% DMTS, 23% DMDS, 9% DM4S | Kite & Hetterscheid, 1997 |
| *A. amygdaloides* Hett. & M. Sizemore | HAM 969 | almond, chemical | 07:00-09:00 | 60% 1-phenylethanone, 40% 1-phenylethyl acetate | Kite & Hetterscheid, 2017 |
| *A. angolensis* (Welw. ex Schott) N.E. Br. | HAM 014 | rotting meat and fish | 15:30-17:30 | 40% limonene, 20% alkanes, 19% DMDS, 18% trimethylamine, 2% DMTS | Kite & Hetterscheid, 2017 |
| *A. angustispathus* Hett. | HAM 1126 | gaseous, sewage/diarrhoea | 06:00-09:00 | 50% isocaproic acid, 22% DMDS, 5% DMTS, 4% 4-methoxyphenethyl alcohol, 4% tetradecane, 3% dodecane | Kite & Hetterscheid, 2017 |
| *A. ankarana* Hett. | HAM 048 | foul | 18:20-21:20 | 39% isoamyl alcohol, 31% 2-nonanone, 8% 1-butanol, 8% 2-nonanol, 3% 2-heptanone, 3% 2-undecanone, 2% 2-decanone | Kite & Hetterscheid, 2017 |
| *A. annulifer* Hett. | unspecified | strong cheese plus rotting meat | 20:15-21:15 | 60% ethyl acetate, 8% DMDS, 7% isoamyl acetate, 5% acetic acid, 2% 2-hepatanone | Kite & Hetterscheid, 1997 |
| *A. antsingyensis* Bogner, Hett. & Ittenb. | HAM 099 | glue, banana | 12:00-14:30 | 43% ethyl acetate, 27% iso/butyl acetate, 19% isoamyl acetate, 2% sobutyl isobutanoate, 2% ethyl propanoate | Kite & Hetterscheid, 2017 |
| *A. aphyllus* (Hook.) Hutch. |  | dung |  |  | Claudel et al., 2017 |
| *A. aphyllus* |  | fruity, melon-like, with added vodka |  |  | pers. commun. S. Jackson |
| *A. atroviridis* Hett. | HAM 683 | spicy cheese | 06:50-09:00 | 98% isocaproic acid, 2% isovaleric acid | Kite & Hetterscheid, 2017 |
| *A. bangkokensis* Gagn. | HAM 1342 | rotting meat | 20:00-22:00 | 76% DMDS, 10% DMTS, 9% ac s-methyl thioesters, 2% 2-methyl-1-propanol, trace elements: DM4S, pr s-methyl thioester, ibu s-methyl thioester | Kite & Hetterscheid, 2017 |
| *A. barthlottii* Ittenb. & Lobin |  | rotting meat |  |  | Moretto et al., 2019 |
| *A. baumannii* (Engl.) N.E. Br. | HAM 666 | rotting meat | 17:30-22:00 | 75% DMDS, 6% isocaproic acid, 5% DMTS, 5% ethyl acetate, 4% DMMS | Kite & Hetterscheid, 2017 |
| *A. beccarii* Engl. | HAM 620 | weak | 19:00-22:00 | 99% 2-methyl-3-buten-2-ol | Kite & Hetterscheid, 2017 |
| *A. borneensis* (Engl.) Engl. & Gehrm. | HAM 158 | rotting meat | 18:30-21:30 | 30% dodecane, 18% DMDS, 10% ethyl acetate, 9% 2-butanone, 8% 3-methyl-1-butanol, 7% 2-pentanone/3-methylbutanone, 3% ac s-methyl thioester, 1% ibu s-methyl thioester, 8% acetone, 3% tetradecane | Kite & Hetterscheid, 2017 |
| *A. boyceanus* Hett. | HAM 515 | gaseous | 17:10-19:10 | 49% DMTS, 39% DM4S, 9% DMDS, trace elements: dimethyl pentasulphide (DM5S) | Kite & Hetterscheid, 2017 |
| *A. brachyphyllus* Hett. | unspecified | fried ﬁsh | 17:00-18:00 | 85% trimethylamine | Kite & Hetterscheid, 1997 |
| *A. brevispathus* Gagn. | HAM 674 | gaseous | 16:00-17:30 | 54% hexane? 17% DMDS, 17% DMTS, 4% 3-methylhexane? | Kite & Hetterscheid, 2017 |
| *A. bulbifer* (Roxb.) Bl. | unspecified | gaseous | 07:30-10:30 | 96% DMTS, 2% DMDS, 1% DM4S | Kite & Hetterscheid, 1997 |
| *A. bulbifer* |  | gaseous |  |  | Punekar & Kumaran, 2010 |
| *A. cicatricifer* Ridl. | unspecified | gaseous plus fruity | 18:05-18-40 | 39% 1-phenylethanone, 24% DMDS, 30% DMTS, 6% 1-phenylethyl acetate | Kite & Hetterscheid, 1997 |
| *A. cicatricifer* | HAM 362 | gaseous, almonds | 05:50-09:10 | 55% 1-phenylethanone, 23% 1-phenylethyl acetate, 12% DMTS, 4% DMDS, 2% dimethyl tetrasulphide, trace elements: 1-phenylethanol | Kite & Hetterscheid, 2017 |
| *A. cirrifer* Stapf (1) | HAM 450 | sewerage, rotting meat | 06:50-12:15 | 46% methyl benzene, 16% isoamyl alcohol, 15% 2/3-methyl-2-butenal; 11% 1-propanol, 6% ethanol; 3% 1-butanol, 2% DMDS | Kite & Hetterscheid, 2017 |
| *A. cirrifer* (2) | HAM 450 | sewerage, rotting meat | 06:45-10:30 | 36% isoamyl alcohol, 20% 1-butanol, 16% 1-propanol, 10% methyl benzene, 3% DMDS, 2% ethanol, 2% 2/3 methylbutanal, 2% 3-methylbutanoic acid | Kite & Hetterscheid, 2017 |
| *A. coaetaneus* Liu & Wie | HAM 672 | gaseous, rotting eggs | 06:00-09:00 | 62% DMDS, 35% DMTS | Kite & Hetterscheid, 2017 |
| *A. commutatus* (Schott) Engl. | HAM 218 | dead meat | 09:30-11:15 | 47% DMDS, 21% ac s-methyl thioester, 11% acetone, 5% DMTS, 3% 2-butanone, 3% 3-methyl-1-butanol; 1% pr s-methyl thioester | Kite & Hetterscheid, 2017 |
| *A. commutatus* |  | rottening meat |  |  | Punekar & Kumaran, 2010 |
| - var*. anmodensis* Sivad. & Jaleel |  | gaseous, fruity |  |  | Punekar & Kumaran, 2010 |
| - var. *anshiensis* Punekar, Lakshmin. & Sivad. |  | gaseous, fruity |  |  | Punekar & Kumaran, 2010 |
| - var*. wayanadensis* Sivad. & Jaleel |  | rottening meat |  |  | Punekar & Kumaran, 2010 |
| *A. consimilis* Bl. (1) | HAM 1150 | glue, banana | 09:00-10:30 | 77% ethyl acetate, 8% propyl acetate, 5% iso/butyl acetate, 2% methyl acetate, 1% isoamyl acetate | Kite & Hetterscheid, 2017 |
| *A. consimilis* (2) | HAM 1150 | glue, banana | 13:00-15:00 | 57% ethyl acetate, 11% propyl acetate, 10% iso/butyl acetate, 7% dodecane, 6% tetradecane, 4% methyl acetate, 3% isoamyl acetate | Kite & Hetterscheid, 2017 |
| *A. corrugatus* N.E. Br. | HAM 082 | gaseous | 06:00-08:00 | 76% DMDS, 20% DMTS | Kite & Hetterscheid, 2017 |
| *A. cruddasianus* Prain ex Engl. | HAM 967 | gaseous | 07:20-08:20 | 54% DMTS, 42% DMDS, 4% DM4S | Kite & Hetterscheid, 2017 |
| *A. dactylifer* Hett. | HAM 226 | rotting meat | 16:30-21:30 | 21% DMDS, 3% DMTS, 25% ac s-methyl thioester, 2% bu s-methyl thioester, 7% ibu s-methyl thioester, 5% butane, 2% 1,8-cineole, 2% decane, 3% linalool, 3% 5-methyl-3-heptanone, 3% 1-octen-3-ol , 6% α-pinene, 2% β-pinene, 4% trimethylamine, trace elements: DMMS | Kite & Hetterscheid, 2017 |
| *A. dunnii* Tutch. | HAM 001 | carrots | 05:30-06:30 | 100% 1-phenylethyl acetate | Kite & Hetterscheid, 2017 |
| *A. dzui* Hett. | HAM 523 | gaseous | 16:00-20:00 | 63% DMDS, 24% DMTS, 10% DM4S, 1% ac s-methyl thioester | Kite & Hetterscheid, 2017 |
| *A. eburneus* Bogn. | HAM 311 | fried ﬁsh | 14:40-15:30 | 64% trimethylamine, 18% acetone, 3% acetic acid, 2% benzaldehyde, 2% DMDS, 2% limonene, 3% nonanal | Kite & Hetterscheid, 2017 |
| *A. eichleri* (Engl.) Hook. f. | unspecified | rotting meat plus dung | 17:30-19:30 | 62% DMDS, 15% DMTS, 1% DM4S, 7% 2-heptanone, 2% indole, 1% phenylethylalcohol | Kite & Hetterscheid, 1997 |
| *A. eichleri* (1) | 1994-7554 | unspecified | 10:30-15:00 | 56% DMDS, 25% 2-heptanone, 8% butyl heptanoate, 7% DMTS, 1% DM4S | Kite & Hetterscheid, 2017 |
| *A. eichleri* (2) | HAM 007 | unspecified | 08:45-09:15 | 30% 2-heptanone, 23% DMDS, 13% 2-pentanone, 10% α-ketoisocaproic acid, 6% 1-butanol, 3% 2-hexanone, 2% 4-methyl-1-pentanol, 2% 2-pentanol, 1% DMTS | Kite & Hetterscheid, 2017 |
| *A. elatus* Ridl. | unspecified | strong cheese | 08:00-10:00 | 100% isocaproic acid | Kite & Hetterscheid, 1997 |
| *A. elliottii* Hook. f. | HAM 1743 | dung | 11:00-13:00 | 35% 3-methylbuten-2-enyl iso/butanoate, 18% 3-methylbut-2-enyl pentanoate/methylbutanoate, 5% 3-methyl-2-buten-1-ol, 3% nonanal, 3% isobutyl isobutanoate, 2% decanal, 2% linalool | Kite & Hetterscheid, 2017 |
| *A. erythrorrhachis* Hett., O. Pronk & R. Kaufmann | HAM 1466 | rotting vegetables | 13:10-14:10 | 56% DMTS, 12% acetone, 7% 2-methyl-1-propanol, 5% DM4S, 4% limonene, 3% α-pinene, 3% sabinene, 2% β-pinene, 2% ac s-methyl thioester, 2% nonanal | Kite & Hetterscheid, 2017 |
| *A. excentricus* Hett. | HAM 867 | gaseous | 06:45-09:30 | 93% DMTS, 5% DMDS, 1% ac s-methyl thioester | Kite & Hetterscheid, 2017 |
| *A. fallax* (Serebryanyi) Hett. & C. Claudel | unspecified | gaseous | 06:45-08:45 | 62% DMTS, 17% DMDS, 15% DM4S, 3% 4-methoxyphenethyl alcohol | Kite & Hetterscheid, 1997 |
| *A. fallax* (1) | 1994-3899 | gaseous, sweet | 10:00-10:30 | 67% DMTS, 10% DMDS, 8% DM4S, 3% 4-methoxyphenethyl alcohol, 1% ac s-methyl thioester | Kite & Hetterscheid, 2017 |
| *A. fallax* (2) | HAM 164 | gaseous, sweet | 06:00-12:30 | 52% DMTS, 24% DM4S, 9% DMDS, 6% 3-hydroxy-2-butanone, 1% DM5S, 1% ac s-methyl thioester, 5% 4-methoxyphenethyl alcohol | Kite & Hetterscheid, 2017 |
| *A. gigas* *Teijsm. & Binnend.* |  | spoiled meat |  |  | Hetterscheid, 1994 |
| *A. gigas* |  | rotten, fishy, sour |  |  | Kakishima et al., 2011 |
| *A. gigas -* appendix sample (1) | unspecified |  | unspecified | 47.3% valeric acid, 35.7% butyric acid, 9.8% acetic acid, 4.1% propionic acid, 3.1% iso-butyric acid | Kakishima et al., 2011 |
| *A. gigas -* appendix sample (2) | unspecified |  | unspecified | 43.8% valeric acid, 27.5% butyric acid, 21.2% acetic acid, 4.4% propionic acid, 3.0% iso-butyric acid | Kakishima et al., 2011 |
| *A. gigas -* spathe sample (1) | unspecified |  | unspecified | 57.4% butyric acid, 22.5% valeric acid, 14.9% acetic acid, 2.9% propionic acid, 2.2% iso-butyric acid | Kakishima et al., 2011 |
| *A. gigas -* spathe sample (2) | unspecified |  | unspecified | 55.2% butyric acid, 22.7% acetic acid, 16.9% valeric acid, 3.3% propionic acid, 1.9% iso-butyric acid | Kakishima et al., 2011 |
| *A. gigas -* spathe sample (3) | unspecified |  | unspecified | 56.9% butyric acid, 19.3% valeric acid, 18.6% acetic acid, 3.0% propionic acid, 2.2% iso-butyric acid | Kakishima et al., 2011 |
| *A. glossophyllus* Hett. | unspecified | gaseous | 07:30-08:30 | 71% DMTS, 20% DMDS, 2% DM4S | Kite & Hetterscheid, 1997 |
| *A. gomboczianus* Pic. Serm. |  | rotting meat |  |  | Gombocz, 1936 |
| *A. haematospadix* Hook. f. | unspecified | bananas | 08:00-11:30 | 65% ethyl acetate, 25% isoamyl acetate | Kite & Hetterscheid, 1997 |
| *A. harmandii* Engl. & Gehrm. | HAM 841 | gaseous | 08:00-09:30 | 64% DMTS, 34% DM4S, 2% DMDS | Kite & Hetterscheid, 2017 |
| *A. henryi* N.E. Br. (1) | HAM 270 | spicy, dung | 12:30-12:40 | 30% isoamyl alcohol, 25% β-pinene, 22% tridecane, 16% α-pinene, 2% camphen, 2% skatole | Kite & Hetterscheid, 2017 |
| *A. henryi* (2) | 1994-3573 | spicy, dung | 15:20-08:40 | 18% isoamyl acetate, 17% 2-butanol, 10% α-pinene, 7% isoamyl alcohol, 7% acetone, 6% tridecane, 6% butyl acetate, 6% isobutyl acetate, 5% undecane, 2% caryophyllene, 2% ethyl acetate, 2% limonene, 2% β-pinene, 1% 3-methyl-2-hexanone | Kite & Hetterscheid, 2017 |
| *A. hewittii* Alderw. | unspecified | ammonia-like |  |  | Chai & Wong, 2019 |
| *A. hirsutus* Teijsm. & Binnend. | HAM 1771 | rotting meat | 11:00-13:00 | 33% DMDS, 17% α-pinene, 11% sabinene, 5% β-pinene, 3% limonene, 3% ac s-methyl thioester, 2% 3-methyl-2-butanone, 2% camphene | Kite & Hetterscheid, 2017 |
| *A. hohenackeri* (Schott) Engl. & Gehrm. |  | foul fetid |  |  | Sivadasan & Sabu, 1989 |
| *A. hottae* Bogn. & Hett. | HAM 914 | faint citrus | 15:30-17:20 | 71% nerolidol | Kite & Hetterscheid, 2017 |
| *A. impressus* Ittenb. | HAM 1384 | rotting vegetables | 05:30-06:30 | 31% aromadendrene, 10% hexadecene, 6% phenol, 3% α-longipinene, 2% caryophyllene, various sesquiterpenoids | Kite & Hetterscheid, 2017 |
| *A. interruptus* Engl. & Gehrm. | HAM 522 | gaseous | 16:00-17:50 | 75% DMDS, 5% DMTS | Kite & Hetterscheid, 2017 |
| *A. johnsonii* N.E. Br. | HAM 1078 | sewerage | 14:00-16:25 | 50% DMDS, 8% 4-methyl-1-pentanol, 7% DMTS, 5% 1,2-dimethylpropyl acetate, 4% isocaproic acid, 3% 3-methyl-2-butanone, 3% ac s-methyl thioester, 2% tetradecane, 2% 2-butanol, 2% dodecane, 1% DMMS | Kite & Hetterscheid, 2017 |
| *A. johnsonii* |  | carrion |  |  | Beath, 1996 |
| *A. julaihii* Ipor, Tawan & P.C. Boyce |  | decomposing shrimp |  |  | Chai & Wong, 2019 |
| *A. konjac* K. Koch | unspecified | rotting meat | 17:30-19:30 | 76% DMDS, 17% DMTS | Kite & Hetterscheid, 1997 |
| *A. konjac* (1) | 1997-111 | rotting meat | 10:00-12:00 | 55% DMDS, 9% ethanol, 6% 2/3-methyl-2-butenal, 6% trimethylamine, 3% 3-methyl-1-butanol, 3% DMTS, 3% acetaldehyle, 2% acetone, 2% 2-butanone, trace elements: DM4S | Kite & Hetterscheid, 2017 |
| *A. konjac* (2) | HAM 168 | rotting meat | 06:00-08:30 | 40% DMDS, 17% DMTS, 12% 2-butanone, 6% acetone, 3% 2/3-methyl-2-butenal, 3% 3-methyl-1-butanol, 2% trimethylamine, 2% 2-methyl-1-butanol, 1% DM4S | Kite & Hetterscheid, 2017 |
| *A. konjac* | China, Kunming Botanical Garden (KBG) | carrion | 10:30-20:30 | 42.9±7.0% DMTS, 26.3±13.4% DMDS, 5.8±1.0% isoamyl alcohol, 3.2±1.1% 2-methyl-1-butanol, 2.5±1.1% β-caryophyllene, 2.3±0.8% 3-methyl-2-pentanone, 2.1±0.9% 4-hydroxy-4-methyl- 2-pentanone, 1.9±0.5% n-nonaldehyde, 1.7±0.4% DM4S, 1.6±1.4% butyl ether, 1.5±0.6% 3-methyl-1-pentanol, 1.3±0.4% n-dodecane, 1.3±0.7% 2-pentanone, 1.1±0.3% n-tridecane, 1.0±1.0% butyl 2-propenoate, 0.8±0.2% n-undecane, 0.8±0.3% decanal, 0.5±0.5% butyl propionate, 0.4±0.4% linalool, 0.3±0.2% humulene, 0.3±0.3% butyl acetate, 0.2±0.2% hexanal, 0.1±0.1% 3-hydroxy-2-butanone, 0.1±0.1% benzyl alcohol | Chen et al., 2015 |
| *A. konkanensis* Hett., Yadav & Patil | HAM 1134 | cheese | 20:20-22:20 | 70% alkanes, 4% S-methyl thioacetate, 4% ocimene, 3% limonene,2% DMDS | Kite & Hetterscheid, 2017 |
| *A. konkanensis* |  | rottening meat |  |  | Punekar & Kumaran, 2010 |
| *A. koratensis* Gagn. |  | gaseous, weakly fruity |  |  | pers. commun. S. Soonthornkalump |
| *A. krausei* Engl. | HAM 040 | gaseous |  | 73% DMTS, 27% DMDS | Kite & Hetterscheid, 2017 |
| *A. lacourii* Linden & Andre | unspecified | gaseous plus anise-like | 07:30-10:10 | 43% DMTS, 28% DMDS, 15% 4-methoxyphenethyl alcohol, 6% DM4S | Kite & Hetterscheid, 1997 |
| *A. laoticus* Hett. | HAM 1012 | human faeces | 20:00-22:00 | 26% ibu s-methyl thioester, 23% ethyl acetate, 20% DMDS, 6% pr s-methyl thioester, 4% ac s-methyl thioester, 4% DMMS, 3% S-methyl-3-methylthiobutanoate, 2% bu s-methyl thioester, 2% DMTS | Kite & Hetterscheid, 2017 |
| *A. lewallei* Malaisse & Bamps | HAM 020 | rotting vegetables | 13:15-15:15 | 86% DMDS, 5% DMMS, 5% DMTS, 4% acetic acid | Kite & Hetterscheid, 2017 |
| *A. linearis* Gagn. | HAM 1164 | cheese | 06:30-10:30 | 94% isocaproic acid, 6% isovaleric acid | Kite & Hetterscheid, 2017 |
| *A. longituberosus* (Engl.) Engl. & Gehrm. | HAM 739 | anise-like | 10:30-12:20 | 98% 4-methoxy- phenethyl alcohol | Kite & Hetterscheid, 2017 |
| *A. macrorhizus* Craib (1) | HAM 990 | strong cheese | 07:00-09:00 | 97% isocaproic acid, 3% isovaleric acid | Kite & Hetterscheid, 2017 |
| *A. macrorhizus* (2) | HAM 1373 | strong cheese | 10:30-11:30 | 95% isocaproic acid, 1% isovaleric acid, 1% acetone | Kite & Hetterscheid, 2017 |
| *A. manta* Hett. & Ittenbach | HAM 347 | cocoa | 06:20-06:55 | 41% DMDS, 18% limonene, 14% DMTS, 7% 1-hexanol, 5% sabinene hydrate, 4% indole, 3% 1-phenylethanol | Kite & Hetterscheid, 2017 |
| *A. margaritifer* (Roxb.) Kunth | unspecified | weak cheese | 16:30-19:30 | 76% ocimene, 7% 1,2-dimethoxybenzene | Kite & Hetterscheid, 1997 |
| *A. maximus* (Engl.) N.E. Br. | unspecified | rotting meat | 13:00-16:00 | 69% DMDS, 21%, DMTS, 4% limonene | Kite & Hetterscheid, 1997 |
| *A. maximus* | HAM 053 | rotting meat, dung | 16:30-17:45 | 75% DMDS, 12% DMTS, 5% cresol, 4% α-pinene, 2% limonene, 1% skatole, trace elements: DM4S | Kite & Hetterscheid, 2017 |
| *A. mossambicensis* (Schott ex Garcke) N.E. Br. (1) | HAM 960 | carrion | 18:00-22:00 | 51% 3-methylbuten-2-enyl iso/butanoate, 38% isobutyl isobutanoate, 3% butyl butanoate, 3% 3-methylbutyl isobutanoate, 3% 3-methylbut-2-enyl pentanoate/methylbutanoate | Kite & Hetterscheid, 2017 |
| *A. mossambicensis* (2) | HAM 448 | carrion | 17:00-20:00 | 75% 3-methylbuten-2-enyl iso/butanoate, 10% 3-methylbut-2-enyl pentanoate/methylbutanoate; 3% isobutyl isobutanoate, 3% isobutyl 2-methylbutanoate, 3% 3-methylbutyl isobutanoate | Kite & Hetterscheid, 2017 |
| *A. mossambicensis* (3) | HAM 447 | acidic, dung | 17:15-18:00 | 54% 3-methylbuten-2-enyl iso/butanoate, 15% 3-methylbut-2-enyl pentanoate/methylbutanoate, 11% isobutyl isobutanoate, 8% isobutyl 2-methylbutanoate, 5% 3-methylbutyl isobutanoate | Kite & Hetterscheid, 2017 |
| *A. muelleri* Bl. | HAM 897 | gaseous, sweet | 06:30-07:45 | unknown % iso/butyl acetate, unknown % DMDS (overloaded chromatographic peak) | Kite & Hetterscheid, 2017 |
| *A. myosuroides* Hett. & A. Galloway | HAM 1644 | sour/acidous | 07:30-09:30 | 75% α-ketoiso-caproic acid, 18% 2,3-heptanedione, 2% isovaleric acid | Kite & Hetterscheid, 2017 |
| *A. napalensis* (Wall.) Bogner & Mayo |  | nauseating, similar to liquid petroleum gas |  |  | Chaturvedi, 2017 |
| *A. napiger* Gagn. | HAM 736 | no observation | 18:00-20:00 | 34% DMDS, 25% DM4S, 23% DMTS, 4% DM5S, 7% 1-hexanol, 5% limonene | Kite & Hetterscheid, 2017 |
| *A. natolii* Hett. *et al*. |  | freshly cut wood |  |  | Hetterscheid et al., 2012 |
| *A. obscurus* Hett. & M. Sizemore | HAM 1544 | fungal, soil | 18:15-20:30 | 21% isoamyl alcohol, 15% 3-undecanone, 15% ethanol, 15% 2-butanone, 10% 1-propanol, 9% 2-methyl-1-propanol, 3% 2-methyl-3-buten-1-ol, 1% 2-pentanone, trace elements: 2-heptanone | Kite & Hetterscheid, 2017 |
| *A. ochroleucus* Hett. & V.D. Nguyen (syn. *A. arnautovii*) | unspecified | spicy | 06:05-07:05 | 74% DMDS, 16% DMTS, 1% limonene | Kite & Hetterscheid, 1997 |
| *A. ongsakulii* Hett. & A. Galloway | HAM 1542 | acidic | 08:00-09:30 | 90% 2-nonanol, 4% 1-methoxynonane | Kite & Hetterscheid, 2017 |
| *A. opertus* Hett. | HAM 143 | gaseous, burnt rubber | 18:00-19:30 | 45% DMDS, 34% DMTS, 10% hexane? | Kite & Hetterscheid, 2017 |
| *A. paeoniifolius* (Dennst.) Nicolson | unspecified | rotting meat | 17:30-19:30 | 47% DMDS, 41% DMTS, 4% DM4S, 4% limonene | Kite & Hetterscheid, 1997 |
| *A. paeoniifolius* | HAM 063 | rotting meat | 16:00-17:30 | 51% DMDS, 33% DMTS, 4% ac s-methyl thioester, 3% DM4S | Kite & Hetterscheid, 2017 |
| *A. paeoniifolius* |  | rottening meat |  |  | Punekar & Kumaran, 2010 |
| *A. pilosus* Hett. | HAM 1089 | rotting meat, sewerage | 17:00-00:20 | 31% 1-butanol, 13% DMDS, 11% 4-methyl-1-pentanol, 10% methoxybenzene, 7% 1-propanol, 6% butenal, 4% DMTS, 3% undecane, 3% hexanal, 2% limonene, 3% 1-pentanol, 2% tridecane, 2% dodecane | Kite & Hetterscheid, 2017 |
| *A. plicatus* Bok & Lam | HAM 1144 | sewerage | 17:15-20:15 | 73% ac s-methyl thioester, 13% trimethylamine, 5% ibu s-methyl thioester, 2% acetone, 2% 4-methyl-1-pentanol, 1% DMMS, 1% DMDS, 1% ethyl propanoate | Kite & Hetterscheid, 2017 |
| *A. polyanthus* Hett. & M. Sizemore (1) | HAM 873 | fungal | 12:30-15:00 | 85% 2-heptanone, 10% hydrocarbons | Kite & Hetterscheid, 2017 |
| *A. polyanthus* (2) | HAM 873 | fungal | 15:30-17:15 | 62% 2-heptanone, 15% heptadecene, 15% pentadecane, 5% 2-nonanone, 2% 2-heptanol | Kite & Hetterscheid, 2017 |
| *A. prainii* Hook f. | unspecified | gaseous | 09:30-11:30 | 65% DMTS, 30% DMDS, 1% DM4S | Kite & Hetterscheid, 1997 |
| *A. prainii* |  | rotten meat |  |  | Soepadmo, 1973 |
| *A. preussii* (Engl.) N.E. Br. | HAM 592 | faintly fruity | 08:30-11:30 | 61% Pyrazine derivative, 19% undecane, 7% dodecane, 5% tetradecane, 2% 4-methyl-2-pentenal | Kite & Hetterscheid, 2017 |
| *A. pulchellus* Hett. & Schuit. | HAM 1581 | sweet | 07:10-19:40 | 30% citronellol, 21% linalool, 6% nerol, 5% pentadecane, 5% tetradecane, 5% 2 acetophenone, 4% β-ionone, 3% hexadecenal | Kite & Hetterscheid, 2017 |
| *A. putii* Gagn. (1) | HAM 1014 | carrots | 06:15-08:45 | 95% 1-phenylethyl acetate, 2% 1-phenylethanone, 1% phenylethene, 1% 1-phenylethanol | Kite & Hetterscheid, 2017 |
| *A. putii* (2) | HAM 971 | carrots | 05:30-09:00 | 100% 1-phenylethyl acetate | Kite & Hetterscheid, 2017 |
| *A. pygmaeus* Hett. | HAM 785 | gaseous | 05:00-07:30 | 35% DMDS, 25% DMTS, 15% DM4S, 10% acetic acid | Kite & Hetterscheid, 2017 |
| *A. sagittarius* | unspecified | gaseous | 07:00-10:00 | 64% DMTS, 28% DMDS | Kite & Hetterscheid, 1997 |
| *A. saraburiensis* Gagn. | HAM 847 | gaseous, cheesy | 16:00-17:15 | 45% DMTS, 23% isocaproic acid, 22% DMDS, 6% DM4S, 4% butanoic acid | Kite & Hetterscheid, 2017 |
| *A. scutatus* Hett. & T.C. Chapman (1) | HAM 589 | gaseous, spicy | 19:30-20:45 | 61% DMTS, 34% DMDS, 5% DM4S | Kite & Hetterscheid, 2017 |
| *A. scutatus* (2) | HAM 590 | gaseous, spicy | 18:15-20:15 | 29% DMTS, 18% DMDS, 12% 1-butanol, 11% DM4S, 7% isocaproic acid, 5% 4-methyl-1-pentanol, 4% butanoic acid, 2% ac s-methyl thioester, 2% acetic acid, 2% ethyl acetate, 2% 2-methylbutanoic acid, 1% DM5S | Kite & Hetterscheid, 2017 |
| *A. sinuatus* Hett. & V.D. Nguyen | HAM 455 | gaseous | 18:00-20:00 | 56% DMDS 33% DMTS, 7% limonene, 2% hexane | Kite & Hetterscheid, 2017 |
| *A. sizemoreae* Hett. | HAM 984 | gaseous | 06:00-08:00 | 72% DM4S, 16% DMTS, 12% DMDS | Kite & Hetterscheid, 2017 |
| *A. sumawongii* (Bogn.) Bogn. (1) | HAM 714 | gaseous, other | 11-30-12:30 | 72% DMTS, 14% DM4S, 9% DMDS, 5% 1-phenylethyl acetate | Kite & Hetterscheid, 2017 |
| *A. sumawongii* (2) | HAM 845 | gaseous, other | 9:15-12:15 | 55% DMTS, 36% DMDS, 4% DM4S, 1% ac s-methyl thioester | Kite & Hetterscheid, 2017 |
| *A. sylvaticus* (Roxb.) Kunth | HAM 1110 | bad vegetables | 05:30-09:30 | 95% 2,6-nonadien-1-ol | Kite & Hetterscheid, 2017 |
| *A. sylvaticus* |  | rottening meat |  |  | Punekar & Kumaran, 2010 |
| *A. symonianus* Hett. & M. Sizemore (1) | HAM 924 | almond, chemical | 16:00-09:30 | 60% 1-phenylethanone, 39% methyl cinnamate, trace elements: phenylethene | Kite & Hetterscheid, 2017 |
| *A. symonianus* (2) | 1998-3421 | almond, chemical | 16:00-16:00 | 89% 1-phenylethyl acetate, 6% methyl cinnamate, trace elements: 1-phenylethanone | Kite & Hetterscheid, 2017 |
| *A. symonianus* | 2017-G-81 | fruity, cinnamon, shoe polish |  |  | personal obs. Cyrille Claudel |
| *A. synandrifer* Hett. & Nguyen V.D. | HAM 1088 | gaseous | 06:00-09:25 | 45% DMTS, 45% DM4S, 8% DM5S, 1% DMDS | Kite & Hetterscheid, 2017 |
| *A. taurostigma* Ittenb. & Hett. | 1994-3548 | sewerage | 19:00-08:00 | 74% butanoic acid, 9% isobutyl, 8% isobutyl butanoate, 4% DMTS, 3% DMDS, 3%-methylbutanoate | Kite & Hetterscheid, 2017 |
| *A. tenuispadix* Hett. | HAM 1244 | anise | 15:00-17:00 | 92% 4-methoxy-phenethyl alcohol, 5% methyl 4-methoxybenzoate | Kite & Hetterscheid, 2017 |
| *A. tenuistylis* Hett. | HAM 772 | pungent and spicy | 19:00-21:45 | 80% 1-phenylethyl acetate, 15% phenylethene, 4% DMDS | Kite & Hetterscheid, 2017 |
| *A. thaiensis* S.-Y. Hu | HAM 945 | citrus smell | 12:30-14:00 | 95% 1-phenylethyl acetate, 5% hydrocarbons | Kite & Hetterscheid, 2017 |
| *A. tinekeae* Hett. & A. Vogel | HAM 830 | ﬁshy | 19:30-21:30 | 35% trimethylamine, 15% decane, 13% ethyl acetate, 9% acetone, 4% DMDS, 2% limonene | Kite & Hetterscheid, 2017 |
| *A. titanum* |  | old fish |  |  | Hetterscheid, 1994 |
| *A. titanum* | unspecified | gaseous plus urine | 18:00-20:00 | 75% DMDS, 10% DMTS | Kite & Hetterscheid, 1997 |
| *A. titanum* | 1997-5514 | gaseous, rotting vegetables | 22:00-07:30 | 70% DMDS, 25% DMTS, 3% ac s-methyl thioester, 1% DM4S, trace elements: DM5S, ibu s-methyl thioester | Kite & Hetterscheid, 2017 |
| *A. titanum* | Palm Garden, Germany | carrion and weakly sweet | 21:35- 23:35 | No DMDS, no DMTS. “Organic compounds with a sharp, pungent smell as well as pleasant ones used for perfumes were found. Benzaldehyde with an almond-like odour dominated the second odour spectrum.” | Lamprecht & Seymour, 2010 |
| *A. titanum* | ex Symon & Hetterscheid | slight rotten fruit like odor, yellow pickled radish, rotten egg, rotting animal-like odor, rotten fish, rotten egg |  |  | Shirasu et al., 2010 |
| *A. titanum* - gas sample |  |  | 21:00-23:00 | (++) trimethylamine, (+) 3-methyl-butanal, (-) methyl thiolacetate, (+) DMTS, (+) acetic acid, (-) isovaleric acid | Shirasu et al., 2010 |
| *A. titanum* - fluid sample |  |  | 01:00-03:00 | (-) trimethylamine, (-) 3-methyl-butanal, (+) methyl thiolacetate, (++) DMTS, (-) acetic acid, (+) isovaleric acid | Shirasu et al., 2010 |
| *A. titanum* | ex Symon & Hetterscheid | decayed cabbage, garlic and pungent sour | unspecified |  | Fujioka et al., 2012 |
| *A. titanum* | Dr Louis Ricciardiello, USA | rotting meat | unspecified |  | Raman et al., 2017 |
| *A. titanum* - appendix sample |  |  | unspecified | 21.6% isovaleric acid, 17.0% butyric acid, 16.2% benzylalcohol, 12.1% γ-butyrolactone, 5.9% 3-hydroxy-2-butanone, 4.4% benzaldehyde, 3.1% 2-phenoxyethanol, 3.0% phenol, 2.9% 4-hydroxy-4-methyl-2-pentanone, 2.4% nonanal, 1.8% trimethyl pyrazine, 0.8% ionol 2, 0.1% 2-ethyl hexanol, 3.1% 2-phenoxyethanol, 3.0% phenol | Raman et al., 2017 |
| *A. titanum* - male flower sample |  |  | unspecified | 27.0% γ-butyrolactone, 13.4% tetradecane, 10.5% 4-hydroxy-4-methyl-2-pentanone, 9.0% ionol 2, 6.1% benzylalcohol, 4.0% isovaleric acid, 3.3% 3-hydroxy-2-butanone, 2.9% phenol, 2.8% α-terpinyl acetate, 2.1% linalool, 2.0% 2-ethyl hexanol, 1.7% 2-phenoxyethanol, 0.1% nonanal | Raman et al., 2017 |
| *A. titanum* - female flower sample |  |  | unspecified | 19.9% tetradecane, 13.0% 4-hydroxy-4-methyl-2-pentanone, 10.6% 3-hydroxy-2-butanone, 8.3% ionol 2, 7.7% 2-ethyl hexanol, 6.9% octacosane, 6.8% heptacosane, 5.1% hexacosane, 4.4% tridecane, 4.3% phenol, 3.8% pentacosane, 3.0% benzylalcohol, 0.1% γ-butyrolactone | Raman et al., 2017 |
| *A. titanum* - spathe sample |  |  | unspecified | 44.5% butyl acetate, 12.2% 3-hydroxy-2-butanone, 5.7% ionol, 2.4% methyl dihydrojasmonate, 2.3% isovaleric acid, 2.3% ionol 2, 2.0% 4-hydroxy-4-methyl-2-pentanone, 2.0% tetradecane, 0.9% benzylalcohol, 0.7% 2-ethyl hexanol, 0.7% 2-phenoxyethanol, 0.5% 6-methyl-γ-ionone | Raman et al., 2017 |
| *A. titanum* |  | rotting flesh, changing to excrement during anthesis |  |  | Giordano, 1999 |
| *A. tonkinensis* Engl. & Gehrm. | HAM 880 | fresh fruit | 07:00-09:15 | 93% 1-phenylethyl acetate, 3% phenylethene | Kite & Hetterscheid, 2017 |
| *A. tuberculatus* Hett. & V.D. Nguyen |  | fresh, candy-like |  |  | Hetterscheid, 2006 |
| *A. variabilis* Bl. (1) | 1997-110 | gaseous | 19:00-09:00 | 73% DMTS, 16% DMDS, 11% DM4S | Kite & Hetterscheid, 2017 |
| *A. variabilis* (2) | 1997-110 | gaseous | 18:10-18:40 | 58% DMTS, 33% DMDS, 7% DM4S | Kite & Hetterscheid, 2017 |
| *A. yuloensis* H. Li (1) | HAM 337 | almond, chemical | 05:30-08:45 | 88% 1-phenylethanol, 11% 1-phenylethanone, trace elements: phenylethene | Kite & Hetterscheid, 2017 |
| *A. yuloensis* (2) | HAM 337 | almond, chemical | 09:15-16:30 | 83% 1-phenylethanol, 7% phenylethene, 6% 1-phenylethanone, 3% benzyl methyl ether | Kite & Hetterscheid, 2017 |
| *A. yunnanensis* Engl. | HAM 874 | carrots | 06:00-08:00 | 100% 1-phenylethyl acetate | Kite & Hetterscheid, 2017 |
